# Advancing thrombopoietin receptor agonist therapy in inherited thrombocytopenia: new insights into avatrombopag use

**DOI:** 10.1016/j.rpth.2026.106955

**Published:** 2026-09-09

**Authors:** Miguel de Oliveira, Sara Morais

**Affiliations:** Unidade Local de Saude de Santo Antonio, Centro de Coagulopatias Congénitas, Unidade de Trombose e Hemostase, Serviço de Imuno-Hemoterapia, Porto, Portugal

The therapeutic landscape of inherited thrombocytopenias (ITs) has evolved substantially with the introduction of thrombopoietin receptor agonists (TPO-RAs). Recently, we reported our experience with eltrombopag in 15 surgical procedures in patients with ITs, with successful responses in 14, reinforcing evidence of its efficacy and safety, particularly in ITs characterized by impaired proplatelet formation [1]. Consistent perioperative responses were observed across multiple IT subtypes, including MYH9-RD, ANKRD26-RT, ACTN1-RT, and ITGA2B/ITGB3-RT, supporting short-term TPO-RA therapy as an alternative to platelet transfusion in selected patients [1].

In his commentary accompanying our work, Zaninetti [2] reinforces a clear message: TPO-RAs should be considered a preferred option for elective surgical preparation in many patients with IT. Their use requires appropriate timing and careful risk–benefit assessment, but they already represent a practical and effective alternative to platelet transfusion.

Importantly, Zaninetti [2] also highlights that a lack of response to one TPO-RA does not preclude a response to another. Switching to an alternative agent may restore platelet responses in nonresponders or partial responders [3,4].

Avatrombopag (AVA), an oral TPO-RA, like eltrombopag, binds to the transmembrane domain of the thrombopoietin receptor [5] and is approved for immune thrombocytopenia and thrombocytopenia associated with chronic liver disease [6]. Its favorable safety profile, particularly the absence of significant hepatotoxicity associated with eltrombopag, makes avatrombopag a plausible alternative in ITs, especially when eltrombopag provides an inadequate response [7].

To our knowledge, only 1 case has been published to date describing the use of AVA in IT, specifically, an MYH9-related disorder initially misdiagnosed as immune thrombocytopenia, in which AVA successfully improved platelet counts following eltrombopag treatment failure [4].

Given the importance of expanding the use of TPO-RAs in ITs, and given the limited evidence available for AVA, we report its use in 3 clinical episodes across 3 patients with ITs, 2 with ANKRD26-RT and 1 with ITGA2B/ITGB3-RT.

The first case involved a 61-year-old man with a long-standing thrombocytopenia of ∼30 years’ duration, caused by a novel likely pathogenic variant in the 5′ untranslated region of *ANKRD26* (c.–125T>C). His usual platelet counts were ∼50 × 10^9^/L. In January 2026, he presented with ecchymosis and petechial lesions at the site of compression from elastic stockings, as well as a costal hematoma following minor trauma, together with a marked worsening of thrombocytopenia (platelet count of 14 × 10^9^/L). Given the reportedly faster platelet response compared with other TPO-RAs [8], AVA was selected and initiated at 20 mg/d. After 6 days, the platelet count increased to 82 × 10^9^/L, accompanied by clear clinical improvement. An AVA taper was promptly initiated: first reduced to 3 times/wk (platelets 94 × 10^9^/L after 1 week), then twice weekly, and subsequently once weekly for an additional 4 weeks. Fifteen days after AVA discontinuation, the platelet count was 24 × 10^9^/L, with no bleeding symptoms.

The second case involves a 78-year-old woman with long-standing familial thrombocytopenia (her mother and sisters also affected), referred for evaluation of thrombocytopenia and perioperative management ahead of total knee replacement. Although from an unrelated family, the same *ANKRD26* variant was identified. After an initial course of AVA 20 mg/d for 1 week, the dose was escalated to 40 mg/d for 14 days, resulting in an increase in platelet counts from 53 × 10^9^/L (66 × 10^9^/L after a week) to 103 × 10^9^/L, which allowed the knee prosthesis surgery to proceed uneventfully.

The third case concerns a young woman who is the daughter of the only patient in our eltrombopag-treated cohort who failed to respond to this agent [1]. She carries a previously reported variant in *ITGA2B* (c.3076C>T) [9]. Her baseline platelet count was 20 × 10^9^/L, notably lower than that of other affected family members [9]. Given her mother’s lack of response to eltrombopag, AVA was selected as the preferred option prior to multiple dental extractions. She started AVA at a dose of 40 mg/d for 14 days, achieving a platelet count of 73 × 10^9^/L. This increase in platelet counts was sufficient to allow the procedure to be performed without complications.

AVA may offer practical advantages over other approved TPO-RAs, including the absence of food or medication restrictions around administration and, importantly, the lack of significant hepatotoxicity [8]. It therefore represents an appealing alternative when a rapid platelet response is desired or when another TPO-RA has failed to provide an adequate response.

Expanding real-world experience with TPO-RAs is essential to better define their role in ITs. As Zaninetti [2] aptly emphasizes, accumulating evidence may eventually support the official approval of TPO-RAs for selected ITs, although this remains a challenging long-term objective.
